# Prevalence and factors associated with chronic low back pain in students of the university of Dschang: a cross-sectional study in a sub-Saharan university

**DOI:** 10.3389/fpain.2026.1743046

**Published:** 2026-02-26

**Authors:** Abdoulaye Abdourahim, Fernando Kemta Lekpa, Sylvain Raoul Simeni Njonnou, Christian Ngongang Ouankou, Sandrine Nadège Chuente Sime, Yannick Fogang Fogoum, Siméon Pierre Choukem, Jerôme Ateudjieu

**Affiliations:** 1 Department of Internal Medicine and Specialities, Faculty of Medicine and Pharmaceutical Sciences, University of Dschang, Dschang, Cameroon; 2 Department of Internal Medicine and Specialities, Douala General Hospital, Douala, Cameroon; 3 Health and Human Development (2HD) Research Network, Douala, Cameroon; 4 Department of Internal Medicine and Specialities, Dschang Regional Annex Hospital, Dschang, Cameroon; 5 Department of Internal Medicine and Specialities, Yaoundé University Teaching Hospital, Yaoundé, Cameroon; 6 Department of Internal Medicine and Specialities, Bafoussam Regional Hospital Centre, Bafoussam, Cameroon; 7 Department of Neurology, Bafoussam Regional Hospital, Bafoussam, Cameroon; 8 Department of Epidemiology and Public Health, Faculty of Medicine and Pharmaceutical Sciences, University of Dschang, Dschang, Cameroon; 9 Ministry of Public Health, Yaoundé, Cameroon; 10 Meilleur Accès aux Soins de Santé (MaSanté), Yaoundé, Cameroon

**Keywords:** chronic low back pain, low back pain, prevalence, student, University of Dschang

## Abstract

**Background:**

Chronic low back pain is a health issue affecting more young people worldwide. This study aimed to determine the prevalence and factors linked to chronic low back pain among students at the University of Dschang.

**Methods:**

We carried out a two-part study focusing on students at the University of Dschang. For the descriptive cross-sectional part, sampling was stratified by faculty and level of study. Participants were chosen systematically, and data were collected through interviews using a pre-tested questionnaire. We calculated the prevalence of chronic low back pain. In the case-control part, students with chronic low back pain served as cases, while those without low back pain were controls. They were matched for age and sex, with one case for every two controls. An adjusted OR was estimated to assess the association between determinants, considering confounding factors, with a 95% CI and assuming *p*-value < 5%.

**Results:**

A total of 1,539 students took part in our study, with a sex ratio of 0.79. The participants’ median age was 20 years [IQR 19–22]. The prevalence of chronic low back pain was 6.2% [95% CI 5.0–7.3]. Most students with chronic low back pain (64.2%) had a mild Roland-Morris disability score (1–6). Being overweight or obese was independently associated with chronic low back pain [ORa = 1.82 (1.02–3.24); *p* = 0.041], as was having a parental history of low back pain [ORa = 2.6 (1.53–4.43); *p* < 0.001].

**Conclusion:**

One in fifteen students at the University of Dschang suffers from chronic low back pain. Being overweight or obese and having a parental history of low back pain were strongly linked to chronic low back pain. Physical exercise and a healthy diet are recommended to help regulate BMI.

## 1 Background

Low back pain (LBP) is widely recognized as one of the most prevalent musculoskeletal conditions and the main cause of years lived with disability worldwide (1, 2). LBP affects individuals of all ages and leads to significant physical, psychological and socioeconomic burdens (1, 3, 4). Although it was traditionally considered to be more prevalent among older adults, recent studies have highlighted its growing prevalence among young individuals, including schoolchildren (5–7). This epidemiological shift has drawn increased attention to the early onset of LBP (1).

In the student population, LBP poses unique challenges. Female gender, obesity, smoking, lifestyle changes, lack of physical activity, parental history of LBP, carrying heavy loads, higher education, academic stress, depressive symptoms, chronic fatigue, prolonged sitting during lectures or study sessions, poor posture, and improper ergonomics characteristics of benches and tables are all suspected to contribute to its development (8–35). If left unaddressed, acute LBP in students can impair academic performance and predispose individuals to lifelong disability and a poor quality of life (8). Despite LBP being increasingly recognized among students, comprehensive data on its prevalence and associated factors remain limited, particularly among those with chronic LBP (CLBP) lasting more than twelve weeks (9–13), and in certain educational contexts and regions, especially in Africa (14–17).

In Cameroon, LBP is believed to affect nearly half of patients seen in Rheumatology clinics (34). A study of 1,075 schoolchildren aged 8–14 years found a prevalence of 12.3% (7). To the best of our knowledge, no research has yet assessed the prevalence of LBP among university students in Cameroon. Furthermore, no published studies on LBP among students in Africa (13–16) have focused specifically on CLBP. Specifically, while some studies conducted among people living with lower back pain have assessed quality of life, to our knowledge, none have been conducted on students with lower back pain (3). One particular issue is the ergonomics of the structures in which students evolve. This study aims to assess the prevalence of CLBP among students and identify socio-demographic, environmental, and clinical factors associated with its onset. By shedding light on these aspects, the study will inform health promotion efforts and policy-making aimed at improving student well-being and academic outcomes.

## 2 Patients and methods

### 2.1 Study design

This was a descriptive cross-sectional survey with a nested case-control component involving students at the University of Dschang. The descriptive component aimed to determine the prevalence of CLBP, which is typically defined as pain in the posterior aspect of the body from the lower margin of the 12th ribs to the lower gluteal folds, with or without pain referred into one or both lower limbs, and lasting more than 12 weeks (1). The case-control component was designed to investigate the determinants of chronic LBP in students at the University of Dschang.

### 2.2 Study site

The study took place on the main campus of the University of Dschang, in the West Region of Cameroon, which has six faculties and two institutes. These are the Faculty of Letters and Social Sciences (FLSS), Faculty of Economics and Management Sciences (FEMS), Faculty of Law and Political Sciences (FLPS), Faculty of Sciences (FS), Faculty of Agronomy and Agricultural Sciences (FAAS), Faculty of Medicine and Pharmaceutical Science (FMPS), and the Fotso Victor's University Institute of Technology (FV-UIT) in Bandjoun and the Institute of Fine Arts in Foumban (IFAF). In 2023, the institution had 19,971 students supervised by several research, teaching, and non-teaching staff.

### 2.3 Duration and period of the study

The study took place from October 2023 to May 2024, i.e., over a period of 1 year and 7 months. Data collection took place between the 1st November 2023 and the 28th February 2024.

### 2.4 Study population and targeted group

The source population was any student regularly enrolled at the University of Dschang, whose faculty is based on the main campus in Dschang, for the 2023–2024 academic year, in the bachelor's or master's cycle.

For the descriptive component, all students who consented to participate in the study were included, and all students who withdrew their consent during the study were excluded.

For the analytical part, the cases were students with CLBP. Students without LBP were also included as controls. Any student who participated in the study and had chronic LBP was included as a case, and any student who participated in the study but did not have LBP was included as a control. Matching was done by age and sex.

### 2.5 Study size

The initial sample size for the descriptive component was 184 participants, calculated using a prevalence of 12.0% among secondary schoolchildren and adolescents, a 95% confidence interval (CI) with a precision of 5%, and a non-respondent rate of 10%. Given that the prevalence of CLBP in our study population is very low, at 12.4% (7, 36). We chose to increase the size of our initial sample. This meant that in order to have 70 cases of chronic LBP for the analytical component calculated using the Schlesselman formula (with Zα/2: 95%; α: 0.05; Zβ of 0.84 for a power of 80% and the ratio = 2:1), we needed a minimum of 688 participants for the descriptive component.

### 2.6 Procedures

After obtaining approval from the Vice-Chancellor of the University of Dschang and the Regional Human Health Ethics Committee, we conducted a random cluster sampling of our participants. First, in all the faculties on the main campus (FLSS, FLPS, FS, FAAS and FMPS), we randomly selected five departments within each of these faculties. The initial contact was made via a telephone call to the department representatives to explain the study and request their assistance with the field visits. We then consecutively visited the rooms (clusters), each representing a single level of study within a specific program in each department, based on their indicated availability. Visits, generally, took place during students' free time or breaks. Within each room, we surveyed as many students as were willing to participate, within the limits of the allocated period. We repeated this process in each faculty until the desired number of students was reached. Participants were asked to complete the questionnaire truthfully and honestly to minimise information bias. Priority was given to the health of the participants.

### 2.7 Data collection tools and variables

An online, well-structured self-report questionnaire was developed based on the results of various pilot articles aligned with our research topic and was validated by medical researchers’ expertise to specifically meet the research objectives. It was also pre-tested. Data was collected through face-to-face interviews, each lasting 15 min. During these interviews, weight and height were measured using the same ultra-slim electronic scale and measuring rod.

The dependent variable was chronic LBP (pain located between the lower margins of the 12th rib and the gluteal folds, persisting for more than 12 weeks) and the independent variables were:

- overweight/obese (BMI greater than 25 kg/m^2^ of body surface),
- physical inactivity (physical activities less than 60 min per day; according to Anses's definition),
- parental history of LBP (history of LBP in one or both parents),
- poor posture in class;
- and poor ergonomics of desks.

The French version of the Roland-Morris Disability Questionnaire – RMDQ (36) was used to assess the functional disability related to CLBP. It comprises 24 questions on the impact of low back pain on physical activities of daily living. Each question corresponds to 1 point, giving a total score of 24 points. The higher the score, the worse the quality of life and the greater the impact.

### 2.8 Statistical analysis

Data were analyzed using IBM Statistical Package for the Social Sciences®, v.20.0. Categorical variables were presented in terms of numbers and frequencies, while quantitative variables were presented in terms of mean ± standard deviation or median [interquartile range] and extremes. The Chi-square test was used to determine the association between the qualitative variables. Associations between low back pain and relevant categorical variables were assessed using the Pearson chi-square test for independence of observations, the Fisher exact test and the Fisher-Freeman-Halton exact test. Associations between low back pain and ordinal variables were performed using the Cochran-Armitage test for trend. Binomial logistic regression and linear regression analyses were employed to assess the independent factors affecting low back pain. Variables at *p* ≤ 0.25 in univariate analysis were entered into multiple logistic regression. The threshold for significance was *p* < 0.05 in multiple logistic analysis.

## 3 Results

### 3.1 Characteristics of the study population

A total of 1,780 students were approached, of whom 1,645 agreed to take part in the study, representing a response rate of 92.4%. However, 106 were excluded for incomplete responses. Finally, 1,539 (93.5%) fully participated in the study and were included in the analysis.

Of the 1,539 students included in the study, 857 (55.7%) were female. The median age was 20 years [9–22], with the extremes of 16–34 years. The 20–24 age group was the most represented, at 58.5%. In terms of faculties and levels, our study population consisted mainly of students from the Faculty of Science, 470 (30.5%), followed by the Faculty of Economics and Management, 298 (19.4%). The Faculty of Medicine and Pharmaceutical Sciences was the least represented, with 123 (8%). The L2 level was the most represented, with 512 (33.3%). The M2 level was in the minority: 119 (7.7%). Almost all the students in our study were of Cameroonian nationality: 1,388 (90.2%). Chad was the most represented foreign nationality, with 142 (9.2%). Almost all of our students were single, 1,511 (98.1%), and only 26 (1.7%) were married. The majority, 821 (53.3%) of our participants, lived in studios (Table 1).

**Table 1:** Participants’ distribution according to socio-demographic characteristics.

Variables	Number (*n*) [*N* = 1,539]	Percentage (%)
Gender		
Female	857	55.7
Male	682	44.3
Age range		
<20	28	29.5
20–24	56	58.9
25–29	9	9.5
>30	2	2.1
Faculties		
FS	470	30.5
FEMS	298	19.4
FLSS	257	16.7
FLPS	242	15.7
FAAS	149	9.7
FMPS	123	8.0
Study level		
L1	421	27.4
L2	512	33.3
L3	352	22.9
M1	135	8.8
M2	119	7.7
Nationality		
Cameroonian	1,388	90.2
Chadian	142	9.2
Gabonese	5	0.3
Ivoirian	3	0.2
Nigerian	1	0.1
Marital status		
Single	1,511	98.1
Married	26	1.7
Widower/widow	1	0.1
Divorced	1	0.1
Type of housing		
In a room	824	53.5
In a family home	485	31.5
In a shared accomodation	152	9.9
In an apartment	78	5.1
University domitory	3	0.2
Presence of electricity at home	1,519	98.7
Presence of flowing water at home	1,168	75.9

### 3.2 Prevalence and distribution of chronic low back pain among students

**Figure 1:**
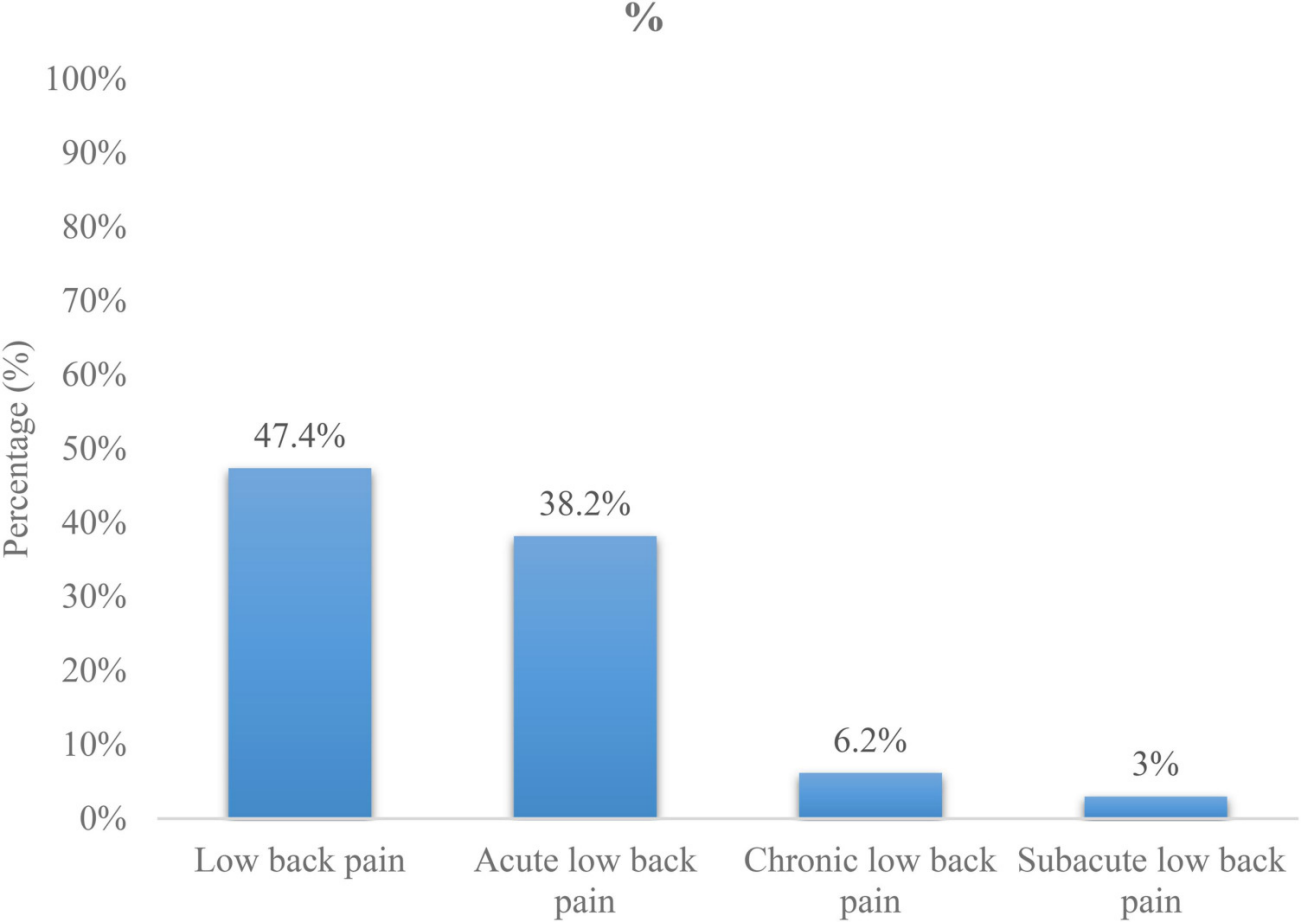
Low back pain prevalence and distribution in the study population.

At the end of our study, 726 of the 1,539 students included, *i.e*., 47.2% [95% CI: 44.7–49.6], had had at least one episode of LBP in their lifetime. Of the 1,539 students included, 95 [6.2% (95% CI: 5.0–7.3)] had CLBP. Five hundred and eighty-five students [38% (95% CI: 35.3–40.5)] had acute LBP, and 46 [3% (95% CI: 2.1–3.9)] had subacute LBP (Figure 1).

Table 2 shows the distribution of students with CLBP by socio-demographic characteristics. The majority of students with CLBP were male (6.3%). Their median age was 21 [19–23] years, with extremes of 17 and 30. According to the faculties, 16 (13%) of the students suffered from LBP in the Faculty of Medicine and Pharmaceutical Sciences.

**Table 2:** Student's distribution suffering of chronic low back pain according to the socio-demographic characteristics.

Variables	Number in a studying population (*n*) *N* = 1,539	Number of students with chronic low back pain (*n*) *N* = 95	Frequency (%)
Gender			
Female	857	52	6.1
Male	682	43	6.3
Age range			
<20	537	28	5.2
20–24	900	56	6,2
25–29	90	9	10
>30	12	2	16.6
Faculties			
FEMS	298	22	7.3
FS	470	20	4.2
FAAS	149	17	11.5
FMPS	123	16	13
FLPS	242	11	4.5
FLSS	257	9	3.5
Study level			
L1	421	11	2.6
L2	512	33	6.4
L3	352	30	8.5
M1	135	15	11.1
M2	119	6	5
Marital status			
Single	1,511	93	6.1
Married	26	2	7.7
Nationality			
Cameroonian	1,388	84	6
Chadian	142	11	7.7
Type of housing			
In a room	824	51	6.1
In a family home	485	30	6.2
In an apartment	78	7	8.9
In a shared accommodation	152	7	4.6
Presence of electricity at home	1,519	95	6.2
Flowing water	1,168	66	5.6

Table 3 shows the distribution of students with chronic LBP according to clinical characteristics and lifestyle. Over 6.4% of the students with poor posture in class had chronic LBP. Thirty-nine (7.5%) of the students taking classes for more than 6 h a day had chronic LBP.

**Table 3:** Student's distribution suffering of chronic low back pain according to their clinical characteristics and their life style.

Variables	Effective in the studying population (*n*) *N* = 1,539	Effective of students with chronic low back pain (n) *N* = 95	Percentage (%)
Sitting position occupied in class			
Poor posture	1,224	78	6.4
Good posture	315	17	5.4
Mean lasting lessons per day			
<6h	1,022	56	5.5
>6h	517	39	7.5
Type of sit			
Bench with backrest	1,214	75	6.1
Bench without backrest	196	16	8.1
Individual chair	122	5	4.1
Type of bag [*N* = 92]			
Hand bag	538	38	7.1
Bagpack	606	30	4.9
Document holder, folder or binder	117	14	11.9
Shoulder bag	208	10	4.8
Physical ability			
No	561	36	6.4
3–4/weeks	847	51	6
4–7/weeks	131	8	6.1
Competitive sports	278	16	5.7
Different competitive sports practiced [*N* = 16]			
Football	169	8	4.7
Basket-ball	28	3	10.7
Gymnastics	16	2	12.5
Athletism	26	1	3.8
Volleyball	21	1	4.8
Tennis	13	1	7.7
Dance	22	1	4.5
Judo	4	1	25
BMI			
Normal BMI	1,065	59	5.5
Overweight	354	25	7.1
Obesity	68	6	8.8
Insufficient mass index	52	5	9.6
Means of transport			
By foot	1,132	70	6.1
By bike	378	25	6.5
Average walking time			
<15 min	186	5	2.7
15–30 min	681	51	7.4
30–45 min	360	24	6.6
45–60 min	179	13	7.3
>1h	127	2	1.6
Usage of stairs	1,168	76	6.5
Place to borrow the stairs *N* = 76]			
Room/House	232	17	7.3
Class room	489	29	5.9
Both	447	30	6.6
Smoking	230	11	4.8
Alcohol consumption			
Always	36	4	10.8
Often	235	9	3.8
Sometimes	338	29	8.6
Rarely	541	40	7.3
Never	383	13	3.4
Past history of low back pains in parents	708	60	8.5
Deformation of the lower limbs	26	1	3.8

BMI, body mass index.

Clinically, weight ranged from 50 to 104 Kg, with a median of 68.1 Kg [60–75]. Of the students with a high BMI, 31 (15.9%) had chronic LBP. And among those with a parental history of LBP, 60 (8.5%) also had chronic LBP.

**Figure 2:**
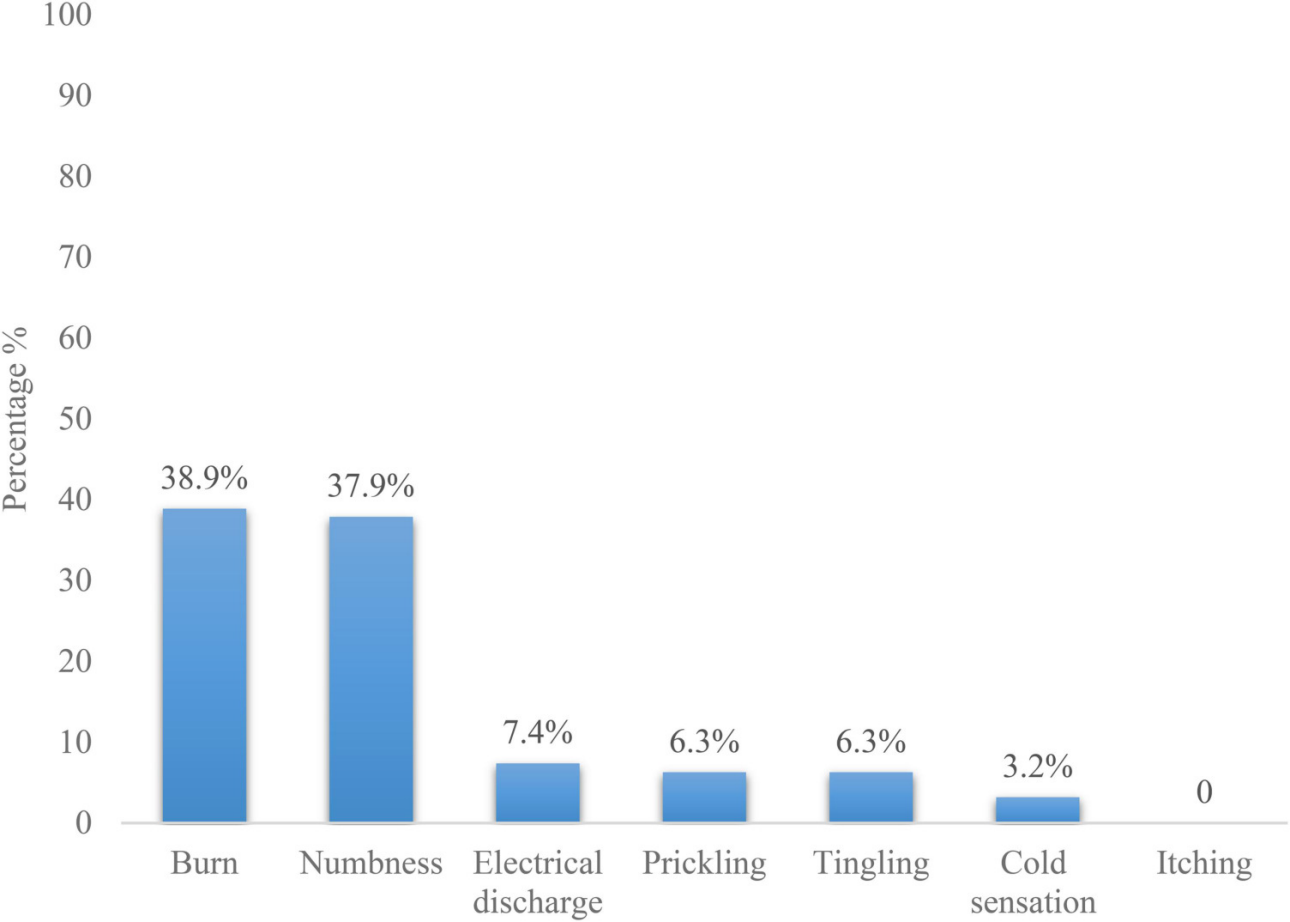
Student's distribution according to the type of Low back pain.

Chronic LBP was expressed in 45.3% of cases as mechanical LBP. It radiated to the buttocks and lower limbs in 20% of cases. The pain was intermittent in 64.2% of cases, permanent in 22.1% and a single episode in 13.7% of cases. Pain was described as burning in 38.9% (*n* = 37) and numbness in 37.9% (*n* = 36) (Figure 2).

The effort of lifting a load was the triggering factor for LBP in 39.5% of cases (*n* = 34). Lying down relieved the pain in 76.7% of cases (*n* = 66). Pain intensity was classified as mild, according to the numerical pain scale (NPS), in 46.2% of cases (Table 4).

**Table 4:** Student's distribution suffering of chronic low back pain according to the intensity of the pains, the releasing factor and aggravating factor.

Characteristics	Number (*n*)	Frequency (%)
Intensity of the pains [*N* = 95]		
None	1	0.1
Mild	336	46.2
Moderate	327	45.0
Severe	63	8.7
[*N* = 86]		
Load lifting efforts	34	39.5
Spontaneously	34	39.5
Flexion and extension	19	22.1
Flexion	15	17.4
Extension	6	7.0
Releaving factors [*N* = 86]		
Lying position	66	76.7
Drugs	24	27.9
Massage at home	23	26.7
Sit position	14	16.3
Physiotherpy	6	7.0

**Figure 3:**
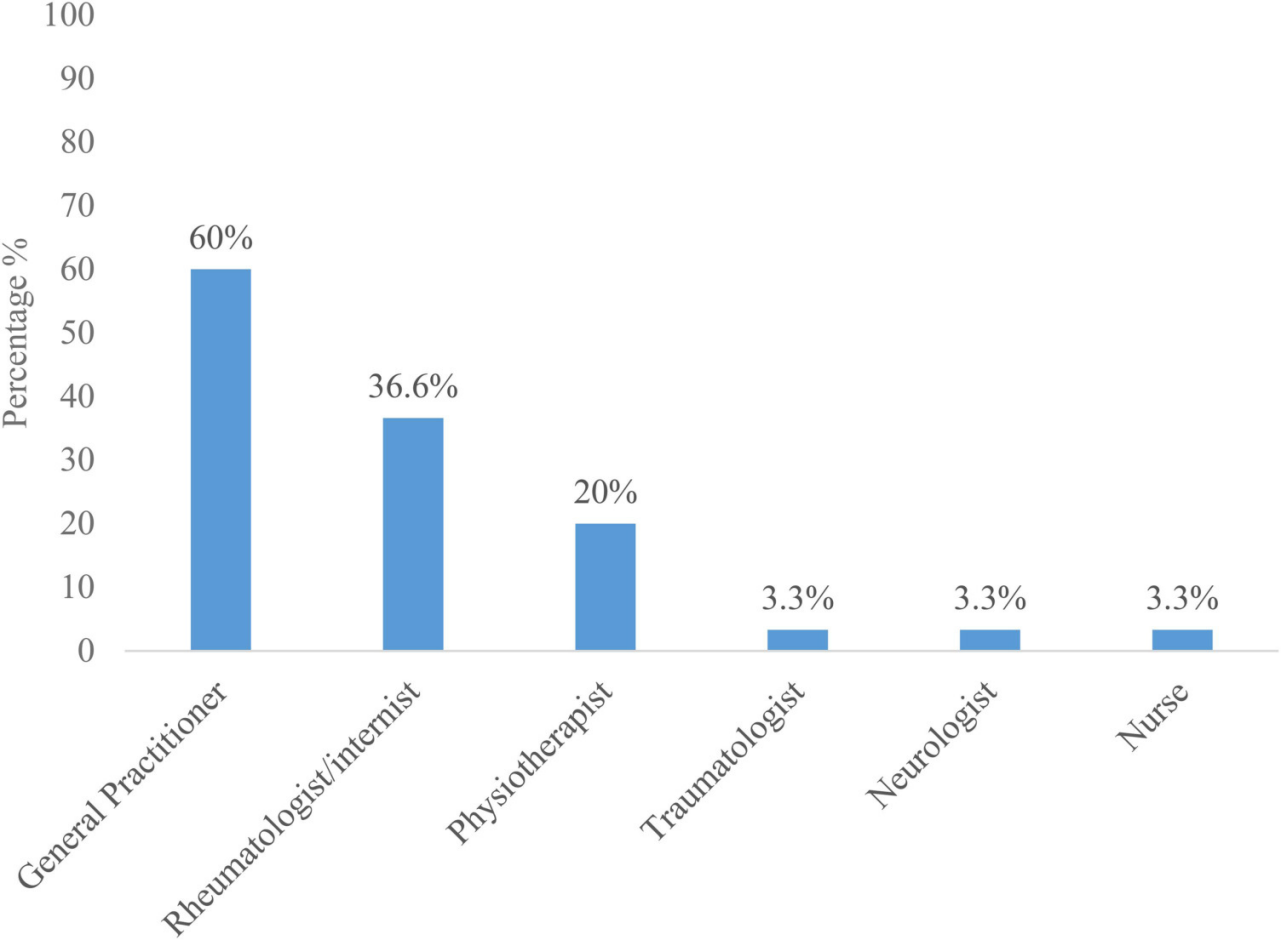
Student's distribution suffering of chronic low back pains according to the health personnel demanded in consultation.

The student was forced to interrupt academic activities in 57.9% of cases. The number of days of interruption of academic activities ranged from 1 day to 38 days, with a median of 3 days [1–4]. Of the 95 students suffering from chronic LBP, 30 (31.6%) had sought medical advice. Of these, 60% had consulted a general practitioner and 36% a rheumatologist (Figure 3).

### 3.3 Quality of life of students with chronic low back pain

Ninety-five students with chronic LBP completed the Roland-Morris questionnaire on quality of life with chronic LBP. The score ranged from 0 to 12, with a median of 4 and an interquartile range of 1–6. Most students with chronic LBP obtained a score of 1–6 (64.2%), followed by 7–14 (20%), then 0 (15.8%). No student obtained a score higher than 14 (Table 5).

**Table 5:** Evaluation of the quality of life style of students with chronic low back pain using the Eifel's score.

Score	Effective (*n*) [*N* = 95]	Percentage (%)
None (0)	15	15,8
Mild (1–6)	61	64,2
Moderate (7–14)	19	20
Severe (>14)	0	0

### 3.4 Factors associated with chronic low back pain in students

Bivariate analysis showed that being overweight/obese (*p* = 0.026) and having a parental history of LBP (*p* < 0.001) were significantly associated with chronic LBP. Overweight or obese students were 1.87 times more likely to have chronic LBP than those with a normal BMI. Students with a family history of LBP were 2.46 times more likely to have chronic LBP.

After adjustment for confounding factors (type of course, parental history of LBP for the overweight/obesity factor; type of course and overweight/obesity for the parental history of LBP factor), being overweight/obese [ORa = 1.82 (95% CI: 1.02–3.24); *p* = 0.041], as well as having a parental history of LBP [ORa = 2.6 (95% CI: 1.53–4.43); *p* < 0.001] remain significantly associated with chronic LBP. Unexpectedly, the following factors were not associated with the development of chronic LBP in our study population: physical inactivity; poor posture in the classroom; and poor ergonomics of bench tables (Table 6).

**Table 6:** Study of factors influencing the occurrence of chronic low back pain, adjusted for confounding factors.

Variables	Chronic low back pain present	Low back pains absent	Crude OR (95% CI)	*P* Unadjusted	Adjusted OR (IC at 95%)	*P* Adjusted
	*n* (%) [*N* = 95]	*n* (%) [*N* = 190]				
Body mass index						
Normal BMI	59 (65.6)	146 (78.1)	Ref	**0**.**026**		
Overweight/obesity	31 (34.4)	41 (21.9)	**1.87** (**1.07–3.26)**		**1.82** (**1.02–3.24)**	**0**.**041**
Parental past history of low back pains						
Yes	60 (63.2)	78 (41.1)	**2.46** (**1.48–4.09)**	**<0**.**001**	**2.60** (**1.53–4.43)**	**<0**.**001**
No	35 (36.8)	112 (58.9)				
Physical activity						
Yes	59 (62.1)	126 (66.3)	0.83 (0.49–1.39)	0.483	0.86 (0.49–1.49)	0.594
No	36 (37.9)	64 (33.7)				
Posture						
Poor	78 (82.1)	146 (76.8)	1.38 (0.74–2.58)	0.307	1.24 (0.64–2.41)	0.521
Good	79 (17.9)	44 (23.2)				
The ergonomics of bench tables						
Poor	16 (16.8)	28 (14.7)	0.85 (0.43–1.66)	0.643	1.06 (0.51–2.23)	0.867
Good	79 (93.2)	162 (85.3)				

Bold values present the association and their degree of variable with CLBP.

## 4 Discussion

The three specifics objectives of this cross-sectional study with a nested case-control component were 1) to determine the prevalence of CLBP, 2) to assess the quality of life of students with CLBP, and 3) to examine whether factors such as overweight/obesity, physical inactivity, parental history of LBP, poor posture, and poor bench ergonomics increase the risk of CLBP among students at the University of Dschang. The current study showed that 47.2% of participants reported LBP, while 6.2% experienced CLBP. Surprisingly, no gender differences were observed among students with CLBP. Of these individuals, two-thirds scored a low score on the Roland-Morris Disability Questionnaire. Only a third of students with CLBP had consulted a healthcare professional about it. Having a parental history of LBP and being overweight or obese were independently associated with CLBP.

To the best of our knowledge, this is the first study to examine the characteristics of CLBP in students in Cameroon. Unlike previous studies, this study specifically evaluated specifically students with CLBP. The prognosis for acute LBP is generally purported to be favourable. However, this is not the case for chronic low back pain, which can lead to disability (1, 2). This survey is also one of the few studies (6, 30–34), not focus solely on health sciences students. Indeed, most published studies on LBP among students have included only medical, nursing and physiotherapy students (8–29). The rigorous random sampling technique employed and the size of the sample obtained are also some strengths of this study. However, this study has some limitations that must be taken into account when interpreting the results. Firstly, due to the cross-sectional design, it is not possible to conclude that there is a causal link between CLBP and BMI or parental history of LBP. Secondly, the single-centre design of the study may limit the generalizability of the results. Nevertheless, this remains possible given that the University of Dschang is a cosmopolitan institution, attracting students from all regions of Cameroon and eleven other African countries. Thirdly, a clinical examination by a physician would have provided a precise medical diagnosis for each student and yielded more qualitative data on their pain experience. This would have enabled us to identify other pain locations, such as neck pain and shoulder pain (4, 28–30). In this study, trained interviewers ensured that the anatomical areas indicated by the students corresponded to the lumbar region. As pain is the main characteristic of LBP, this survey is sufficient for an accurate LBP diagnosis. Fourthly, we did not sufficiently consider the psychological aspects of LBP in students. It was assessed indirectly using the RMDQ (35), designed to measure the impact of CLBP on patients' daily activities. However, we did not find a significant association between these factors and CLBP. Further studies assessing the clinical, radiological and psychological characteristics of CLBP in students in SSA would be interesting. Finally, it would be interesting to conduct a follow-up study with these students to assess how their low back pain evolves during their professional lives.

Many studies assessed LBP in Cameroon before. However, most of them targeted either healthcare workers or schoolchildren. None of them specifically targeted students or investigated the impact on quality of life. Unlike the data found in older people, there is no difference between men and women among students. The main factors identified are overweight/obesity and a family history of LBP. However, we did not explore the impact of stress and anxiety on CLBP (7, 37, 38).

Based on our data, and despite the limitations of the study, policymakers should consider CLBP as a public health issue affecting also young adults, including students. Risk factors significantly associated with CLBP (8–34), particularly the factors identified in our study, should be incorporated into algorithms for the prevention and treatment of LBP. Ergonomic facilities should be included in ergonomics programs developed for students, taking into consideration the type of training. In this study, the level of disability was mild, but this was in contrast to the high number of students who had interrupted their academic activities due to LBP compared to data from other countries (9). Previous research shows that fewer than one in four students with low back pain seek formal medical advice, highlighting possible barriers to accessing healthcare or a low perception of the severity of the condition (16, 19, 21, 33). The high consultation rate observed in our study could be explained by the fact that all University of Dschang students have health insurance, and by the proximity of two university hospitals staffed by experienced medical professionals. The reduced impact on quality of life observed in our study could be explained by rapid and effective treatment.

Further rigorous studies should be conducted to assess the impact of the triggering or aggravating factors of LBP identified, such as carrying heavy loads, flexion and extension movements, long sitting sessions, stress and poor sleeping position (11, 25). Similarly, investigating the effect of weight reduction on the prevention of LBP could help to establish a cause-and-effect relationship between CLBP and BMI.

## 5 Conclusion

CLBP is common in university settings. It affects one in fifteen students in a Cameroonian university. However, CLBP does not significantly impact the quality of life of these students. Factors associated with CLBP in this study were parental history of LBP and overweight/obesity. However, physical inactivity, poor posture in class, and poor bench ergonomics were not associated with CLBP in this study. We suggest that preventive measures, such as ergonomic facilities and psychosocial supports, must be taken in young adults, as in childhood, to prevent the occurrence of CLBP and its persistence into adulthood. Our findings support the need for further research to improve the understanding of LBP in students.

## Data Availability

The original contributions presented in the study are included in the article/Supplementary Material, further inquiries can be directed to the corresponding author.
